# Stability of a Compressed Bar Resting on an Elastic Substrate with Stepwise Changes in Parameters

**DOI:** 10.3390/ma19020258

**Published:** 2026-01-08

**Authors:** Mirosław Sadowski, Jakub Marcinowski, Volodymyr Sakharov

**Affiliations:** 1 Independent Researcher, 59-900 Zgorzelec, Poland; miroslaw.sadowski@gmail.com; 2 Independent Researcher, 65-191 Zielona Gora, Poland; j.marcinowski@ib.uz.zgora.pl; 3 Institute of Building Engineering, University of Zielona Gora, Campus A, 65-516 Zielona Gora, Poland

**Keywords:** compressed bar, variability of elastic foundation parameters, analytical solution, numerical simulation, mathematica, COSMOS/M, ABAQUS

## Abstract

The study presents a stability analysis of an axially compressed column resting on a Winkler foundation with a stepwise variation in stiffness. The solution is based on an energy approach using the Rayleigh quotient, and the original buckling mode function is proposed to capture the localization of deformations in the region of foundation discontinuity. The theoretical model was verified numerically for rectangular-section columns by comparing the results with simulations performed in COSMOS/M and ABAQUS systems. The differences in critical load values did not exceed 1.7%. The investigation showed that increasing the stiffness contrast leads to stronger buckling localization within the weaker foundation segment. The developed model can be used for preliminary assessment of the load-carrying capacity of structural elements interacting with a non-homogeneous distributed foundation.

## 1. Introduction

The problem of interaction between compressed members and an elastic medium has a long history, encompassing linear, nonlinear, and non-homogeneous models. The foundation of this field is the Winkler theory [1], which initiated analyses of elements supported by an elastic foundation. At the turn of the 19th and 20th centuries, the topic was advanced by Engesser [2], Jasiński [3,4,5], Timoshenko [6,7], Bubnow [8], Bleich [9], Ratzersdorfer [10], Papkowicz [11], Snitko [12], and Lejtes [13], who applied various computational methods and examined the influence of the spacing and stiffness of elastic supports. A major step was the comprehensive theory of beams on a Winkler foundation developed by Hetényi [14]. It included the effective range of loading and provided a wide set of analytical solutions forming the basis of later models. Pasternak [15] extended the classical model to a two-parameter foundation, and Vlasov and Leontiev [16] generalized the theory to beams, plates, and shells. Volmir [17] further developed the buckling theory of members on elastic foundations and flexible supports, accounting for the effect of foundation stiffness on critical load, buckling modes, and dynamic behavior, and highlighting applications in thin-walled structures. In subsequent decades, research focused on nonlinear and post-buckling behavior. Teodorescu [18] showed that increasing foundation stiffness leads to a nonlinear rise in critical load and alters buckling characteristics. Kounadis, Mallis, and Sbarounis [19] expanded this topic into the post-buckling regime, analyzing the influence of foundation properties and geometric nonlinearity. In the second decade of the 21st century, studies included advanced models: Obara [20] presents a very important question of relationships between the parameters describing the beam vibration, the compressive force and the foundation parameters; Tossapanon and Wattanasakulpong [21] investigated layered beams with variable material properties; and Ghadban [22] developed a general method for analyzing non-prismatic columns on foundations of variable stiffness. Deniziak and Iwicki [23] examined cold-formed C-section columns with elastic lateral restraints, demonstrating the relationship between support stiffness and buckling mode, while Baláž, Koleková, and Moroczová [24] analyzed multi-span members with point elastic supports, relevant particularly to bridge engineering. More recent studies focus on non-homogeneous, variative, and computationally complex foundations. Abramian, Vakulenko, and van Horssen [25] investigated vibrations and buckling of beams on non-homogeneous foundations with damping; Loya, Santiuste, Aranda-Ruiz, and Zaera [26] analyzed cracked columns, Kanwal et al. [27] demonstrated asymmetry (different behavior in adjacent segments) of buckling modes caused by variability of the Winkler coefficient and Zhang, Hu and Chen [28] analyzed the stability of a compression–bending bar embedded in a viscoelastic Winkler-type medium, showing that the actual properties of the elastic foundation—particularly the spatially varying reaction coefficient—exert a decisive influence on the displacements and load-bearing capacity of the bar. Ike [29] developed a critical-load model for beams on two-parameter foundations, while Thang, Tien, and Thuan [30] applied FEM to columns with varying geometry. In the same period, Xu et al. [31] proposed an improved Winkler modulus accounting for the material properties of both beam and foundation. Filippov [32] conducted analytical and numerical investigations of shear-wave propagation in a thin elastic plate with a hole resting on a rough base. The obtained solutions demonstrate that the contact conditions between the plate and the foundation may lead to complex equilibrium states and significant variations in residual stress distributions. Posso, Molina-Villegas, and Ballesteros Ortega [33] introduced the GFSM method for Timoshenko beams and frames, enabling accurate analytical solutions. Previati et al. [34] presented a numerical method which, thanks to variable reduction, enables fast and accurate modeling of beams on nonlinear elastic foundations and Indroil and Nazmul [35] introduced innovative developments of elastic foundation models using the mathematical properties of integral convolution. Krutii et al. [36] analyzed dynamic mode shapes of beams on non-homogeneous foundations. Significant contributions were also made by Wstawska, Magnucki, and Kędzia [37] and by Magnucki [38], who examined buckling of members on foundations with variable stiffness characteristics. Contemporary research covers both the development of foundation parameters and numerical improvements. In the works of Xu et al. [31], Posso, Molina-Villegas and Ballesteros Ortega [33], and Previati et al. [34], attention was focused on more accurate representation of foundation parameters, new computational approaches, and refined analytical methods. At the same time, the influence of local stiffness discontinuities on buckling shape and localization remains a key research challenge. Artan and Tepe [39] demonstrated that accurate modeling of the elastic foundation is essential for correctly describing the static behavior of curved nanobeams within a nonlocal elasticity framework. Ahmed, Zakria, Osman, Suhail and Rabih [40] showed that the two-parameter viscoelastic Pasternak foundation significantly modifies wave propagation and attenuation in nonlocal thermoelastic microbeams. Uzun [41] reported that the elastic stiffness and shear modulus of the Pasternak foundation have a substantial effect on the vibration characteristics of short-fiber-reinforced composite microbeams. Hafed and Zenkour [42] found that the parameters of the Winkler–Pasternak foundation distinctly alter the natural frequencies and dynamic response of a thermoelastic microbeam.

In this context, the originality of the present study lies in formulating a model for an axially compressed member resting on an elastic foundation with stepwise stiffness variation, accounting for local deformation behavior in the region of stiffness discontinuity. The model is based on an energy method using the Rayleigh quotient, enabling analytical estimation of the critical load. An original buckling-mode function was introduced, incorporating parameters describing the asymmetry of the deflection shape and the sharpness of the transition between regions with different foundation stiffness. This approach makes it possible to capture local elastic effects that classical models with continuous stiffness distribution fail to represent.

**Figure 1:**
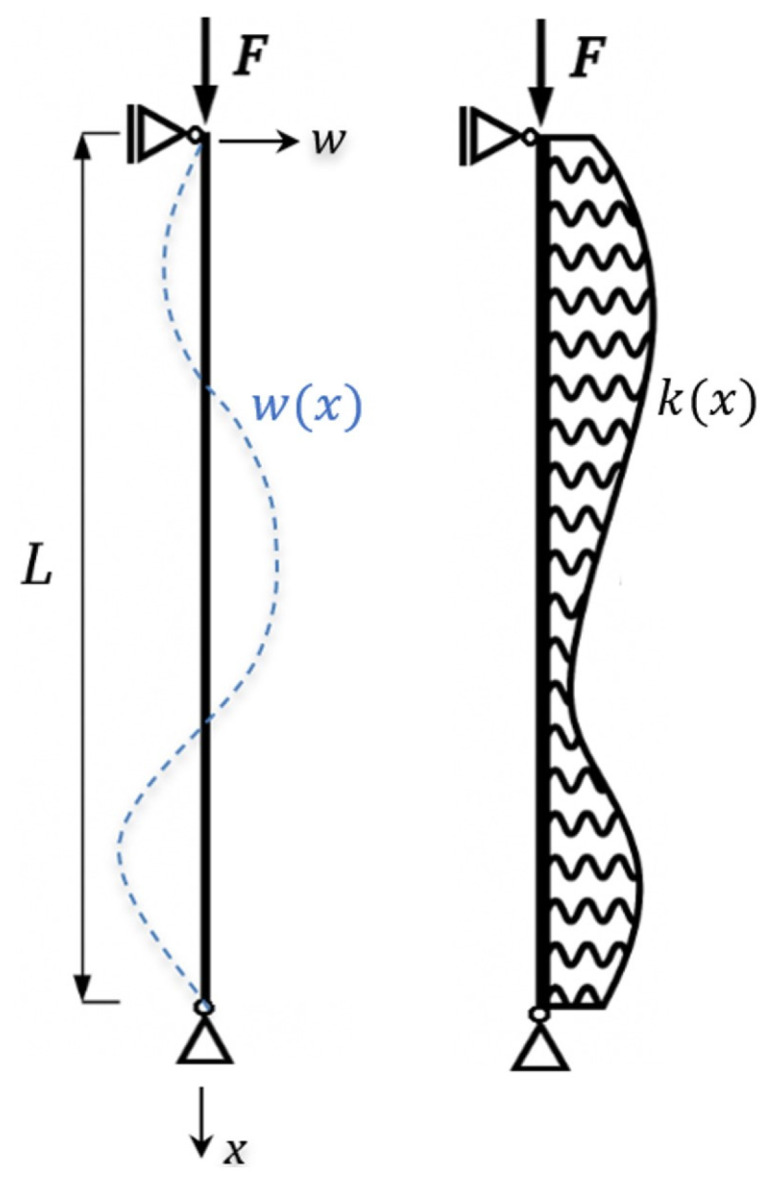
The bar under consideration, together with the buckling form $w=w\left(x\right)$ and a variable coefficient of elasticity of the substrate $k=k\left(x\right)$.

**Figure 2:**
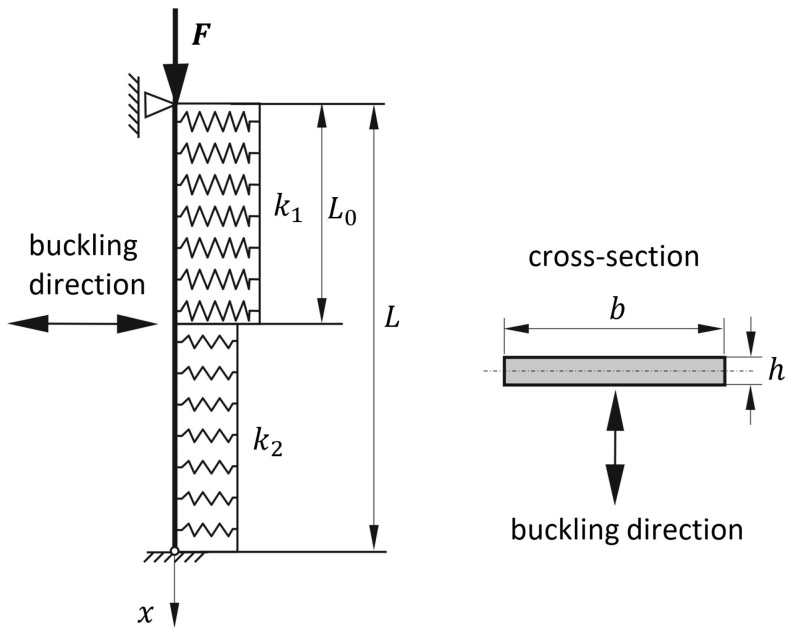
A bar with a stepwise variable coefficient of elasticity of the substrate.

The objective of the present work is to analyze the behavior of prismatic axially compressed members with pinned ends, placed in an elastic medium whose stiffness changes stepwise along the length (Figure 1 and Figure 2).

## 2. Buckling of a Simply Supported Bar Compressed by Axial Force and Placed in an Elastic Medium

Let $w=w\left(x\right)\;$ denote the deflection of a bar hinged at both ends, whose length is L  (Figure 1). Let $k=k\left(x\right)$ expresses the elasticity of the medium in which the bar is located. The origin of the coordinate system is located at the upper end of the bar (Figure 1). Due to the layout of the system, the assumed deflection of the bar satisfies the boundary ${\left[w\right]}_{0}^{L}=0$. The symbol ∫…dx is understood to mean $\int_{0}^{L}\ldots dx$.

### 2.1. Rayleigh Quotient

The energy of elastic bending deformations of the column and the elastic energy of the elastic substrate shown in Figure 1 are described by the functional (Timoshenko and Gere [43], Vlasov and Leontiev [16]):

(1) $$U\left[w\right]=\frac{EI}{2}\int {\left(w\prime \prime (x)\right)}^{2}dx+\frac{1}{2}\int k{\left(x\right)w}^{2}\left(x\right)dx,$$

where E expresses the Young’s modulus, I—the moment of inertia of the cross-section, $k\left(x\right)$—the Winkler subgrade elasticity coefficient, while $w\left(x\right)$ denotes the postulated form of bar buckling.

The derivation of relation (1) is based on the following reasoning:

✓ the curvature of the deformed axis of the beam can be approximated by $\kappa (x)\approx w^{\prime \prime }(x)$, and the bending moment is given by $M(x)\approx EI\;w^{\prime \prime }(x)$. The elemental bending strain energy is, by definition, $\;dU_{g}=M^{2}(x)/\left(2EI\right)\;dx,$ which leads to $dU_{g}=1/2\;EI\;(w^{\prime \prime }(x){)}^{2}\;dx.$ Therefore, the total bending strain energy becomes $U_{g}[w]=EI/2\int {\left(w\prime \prime (x)\right)}^{2}dx$,
✓ in the Winkler foundation model, the reaction per unit length satisfies q(x)=k(x)w(x), where k(x) denotes the local foundation stiffness. Assuming linear elasticity, the elemental strain energy of the foundation corresponding to an infinitesimal segment dx  is $\;dU_{k}(x)=1/2\;k(x)\;w^{2}(x)dx$ and therefore $U_{k}[w]=1/2\int k(x)w^{2}(x)\;dx,$
✓ finally, the total potential energy of the system is $U\left[w\right]=U_{g}\left[w\right]+U_{k}\left[w\right].$

The work performed by the axial force F resulting the transverse displacement $w\left(x\right)$ can be written in the form (Volmir [17]):

(2) $$V\left[w\right]=\frac{F}{2}\int {\left(w\prime (x)\right)}^{2}dx.$$

Relation (2) is obtained as follows: the axial force F acts along the beam and remains constant. The length of an infinitesimal beam element in the deformed configuration is $ds=\sqrt{1+(w^{\prime }(x){)}^{2}}\;dx\approx 1+1/2(w^{\prime }(x){)}^{2})dx.$ Hence, the corresponding extension is $\Delta s=ds-dx\approx 1/2(w^{\prime }(x){)}^{2}dx.$ The elemental work of the axial force is therefore $dV=F\;\Delta s=F/2\cdot (w^{\prime }\left(x\right){)}^{2}dx.$ Integration along the length of the beam yields relation (2).

Critical equilibrium occurs when the increase in elastic energy is equal to the increase in work performed by an external force, i.e., when U[w]=V[w], meaning that

$$\frac{EI}{2}\int {\left(w\prime \prime (x)\right)}^{2}dx+\frac{1}{2}\int k{\left(x\right)w}^{2}\left(x\right)dx=\frac{F}{2}\int {\left(w\prime (x)\right)}^{2}dx,$$

from which the critical force value can be obtained in the form of the so-called Rayleigh quotient (Hetényi [14]):

(3) $$F=\frac{EI\int {\left(w\prime \prime (x)\right)}^{2}dx+\int k{\left(x\right)w}^{2}\left(x\right)dx}{\int {\left(w\prime (x)\right)}^{2}dx},$$

Expression (3) determines the approximate value of the critical force for the assumed form of buckling w(x) and is the basis for many classical energy analyses of beam-support systems (Timoshenko and Gere [43]; Obara [20]).

### 2.2. Analysis of System Behavior and Assumption of Buckling Form

Let us consider a bar placed in an elastic medium of the form

(4) $$k\left(x\right)=\left\{\begin{array}{c} k_{1},\;\;\;for\;\;\;0\leq x\leq L_{0},\\ k_{2},\;\;\;for\;\;\;L_{0}\leq x\leq L, \end{array}\right.$$

where $0<L_{0}<L$ or, in standardized form (Figure 2)

(5) $$k\left(\xi \right)=\left\{\begin{array}{c} k_{1},\;\;\;for\;\;\;0\leq \xi \leq \xi_{0},\\ k_{2},\;\;\;for\;\;\;\xi_{0}\leq \xi \leq 1, \end{array}\right.$$

where

(6) $$\begin{array}{cc} \xi =x/L, & \xi_{0}=L_{0}/L \end{array}.$$

For the adopted arrangement, let the buckling form be

(7) $$w\left(x\right)=A\sin\frac{m\pi x}{L}\left(1+\alpha \tanh\gamma \left(x-\frac{L_{0}}{L}\right)\right),$$

where 0≤x≤L,  A—amplitude of buckling mode, m∈Z,α∈R,γ∈R.

Relation (7) can be defined in a dimensionless form

(8) $$w\left(\xi \right)=A\sin m\pi \xi \left(1+\alpha \tanh\mu \left(\xi -\xi_{0}\right)\right),$$

where 0≤ξ≤1, $0\leq \xi_{0}\leq 1,$ μ=γL.

The buckling mode was chosen as the product of a sinusoidal function and a hyperbolic tangent. Such a construction captures the local deformation behavior in the region of the foundation stiffness jump. The sinusoidal factor provides the correct global modal shape, consistent with classical buckling theory, ensuring the kinematic conditions w(0)=w(L)=0  and enabling control of the number of half-waves through the parameter m. The hyperbolic factor, in turn, acts as a modulating function that introduces different behavior in adjacent segments and controls localization of deflection near the stiffness-change point, reflecting the numerically observed shift in maximum deflection toward the weaker segment. This trial function combines analytical simplicity with the ability to model effects unattainable using the classical sinusoidal form alone, making it particularly useful for problems involving stepwise stiffness discontinuities in the foundation.

The parameter m present in the relation (8) governs the number of half-waves and allows the representation of both the fundamental and higher buckling modes, which is essential in systems with strong stiffness contrasts. In systems with a stepwise variation in the Winkler modulus, the classical sinusoidal function sin (πx/L), suitable for homogeneous foundations, does not reproduce the deformation localization nor the asymmetry of the buckling shape. Introducing the parameter α makes it possible to shift the maximum deflection toward the weaker segment of the foundation, whereas the parameter μ controls the “sharpness’’ of the transition at the point where k(x) jumps. Small values of μ lead to smooth transitions, while large values produce an abrupt change in curvature, consistent with numerical results.

From the viewpoint of the Rayleigh–Ritz method, expression (8) serves as a trial function and therefore does not need to satisfy all boundary conditions. This is admissible because, in the Rayleigh method, the approximate critical load corresponds to the value of the quotient (3). By the classical Courant–Fischer theorem, any admissible trial function yields $F_{approx}\geq F_{cr}$, i.e., the result is an upper bound on the first eigenvalue associated with the true buckling mode. The accuracy, however, depends on how well the trial function approximates the actual eigenmode. For the present function (8), the inaccuracy arises from the fact that the displacement conditions w(0)=w(L)=0 are satisfied, while the conditions $w^{\prime \prime }(0)=w^{\prime \prime }(L)=0$ are not. As a consequence, the assumed curvature near the beam ends deviates from the exact one, leading to a local overestimation of the bending strain energy (the assumed $w^{\prime \prime }(x)$ near the boundaries does not coincide with the exact curvature). In general, overestimating the curvature and bending moments near the ends leads to an excessive bending energy, and consequently to an overestimation of the Rayleigh quotient. Therefore, the error can be described qualitatively but not explicitly quantified. Eigenvalue theory nevertheless asserts that the error diminishes as the trial function more closely resembles the true critical mode. Furthermore, the error is concentrated in the regions where boundary conditions are not met (here: the beam ends). The inclusion of the parameters α and μ improves accuracy by enabling a better fit of the mode shape in the vicinity of the stiffness discontinuity.

Thus, the buckling function (8) combines analytical simplicity with the ability to accurately reproduce deflection localization, different behavior in adjacent segments, and the deformation character in systems with stepwise variable foundation stiffness, thereby satisfying the standard requirements of the energy method.

The detailed significance of buckling mode parameters:

✓ m—number of half-waves along the length of the bar (integer),
✓ α —asymmetry parameter (sign and strength of buckling location):
  - α>0: an amplitude in the upper half of the system,
  - α<0: amplitude in the lower half of the system,
  - α=0: symmetrical shape,
✓ μ—‘transition sharpness’ parameter (nature of the change in form at the point of compression change):
  - low μ value: gentle, blurred transition,
  - high μ value: sharp transition exactly in the middle of the rod.
  - Let us implement the form of buckling (8) and the type of elastic medium (5) in the Rayleigh quotient (3). Due to (8),

$$\frac{dw}{d\xi }=A\left[m\pi \cos m\pi \xi \left(1+\alpha \tanh\mu \left(\xi -\xi_{0}\right)\right)+\alpha \mu \sin m\pi \xi {\mathrm{sech}}^{2}\mu \left(\xi -\xi_{0}\right)\right],$$

d2wdξ2=A−mπ2sinmπξ1+αtanhμξ−ξ0+2αμmπ cosmπξsech2μξ−ξ0−2αμ2sinmπξtanhμξ−ξ0sech2μξ−ξ0

and

$$\frac{dw}{dx}=\frac{dw}{d\xi }\cdot \frac{d\xi }{dx}=\frac{1}{L}\frac{dw}{d\xi },\frac{d^{2}w}{dx^{2}}=\frac{d}{dx}\left(\frac{dw}{dx}\right)=\frac{1}{L}\frac{d}{d\xi }\left(\frac{1}{L}\frac{dw}{d\xi }\right)=\frac{1}{L^{2}}\frac{d^{2}w}{d\xi^{2}}.$$

Furthermore,

$$EI\int {\left(d^{II}w\right)}^{2}=EI\int_{0}^{L}{\left(\frac{d^{2}w}{dx^{2}}\right)}^{2}dx=EI\int_{0}^{1}{\left(\frac{1}{L^{2}}\frac{d^{2}w}{d\xi^{2}}\right)}^{2}Ld\xi =\;\;\;=EI\int_{0}^{1}\frac{1}{L^{4}}{\left(\frac{d^{2}w}{d\xi^{2}}\right)}^{2}Ld\xi =\frac{EI}{L^{3}}\int_{0}^{1}{\left(\frac{d^{2}w}{d\xi^{2}}\right)}^{2}d\xi ,$$

$$\begin{array}{c} \int kw^{2}=k_{1}\int_{0}^{L_{0}}w^{2}\left(x\right)dx+k_{2}\int_{L_{0}}^{L}w^{2}\left(x\right)dx\\ \;=k_{1}\int_{0}^{\xi_{0}}w^{2}\left(\xi L\right)Ld\xi +k_{2}\int_{\xi_{0}}^{1}w^{2}\left(\xi L\right)Ld\xi =\\ \;=L\left(k_{1}\int_{0}^{\xi_{0}}w^{2}\left(\xi L\right)d\xi +k_{2}\int_{\xi_{0}}^{1}w^{2}\left(\xi L\right)d\xi \right), \end{array}$$

$$\int {\left(dw\right)}^{2}=\int_{0}^{L}{\left(\frac{dw}{dx}\right)}^{2}dx=\int_{0}^{1}{\left(\frac{1}{L}\frac{dw}{d\xi }\right)}^{2}Ld\xi =\int_{0}^{1}{\frac{1}{L^{2}}\left(\frac{dw}{d\xi }\right)}^{2}Ld\xi =\frac{1}{L}\int_{0}^{1}{\left(\frac{dw}{d\xi }\right)}^{2}d\xi .$$

Substituting the last relationships into the Rayleigh quotient, we obtain

(9) $$\begin{array}{c} F\left(m,\alpha ,\mu \right)=\frac{\frac{EI}{L^{2}}\int_{0}^{1}{\left(\frac{d^{2}w}{d\xi^{2}}\right)}^{2}d\xi +L^{2}\left(k_{1}\int_{0}^{\xi_{0}}w^{2}\left(\xi L\right)d\xi +k_{2}\int_{\xi_{0}}^{1}w^{2}\left(\xi L\right)d\xi \right)}{\int_{0}^{1}{\left(\frac{dw}{d\xi }\right)}^{2}d\xi }. \end{array}$$

Determining the critical force requires minimizing the functional (9), i.e.,

(10) $$F_{cr}=\underset{m,\alpha ,\mu }{\min}F\left(m,\alpha ,\mu \right).$$

## 3. Numerical Examples

To verify the theoretical model, a computational analysis was carried out for three sets of geometric and material parameters of the bar, as well as for various configurations of the elastic foundation properties. The adopted cases included different ratios of foundation stiffness, which made it possible to examine the influence of stiffness contrast on the value of the critical force and the shape of the buckling form. In each variant, a bar hinged at both ends, with a rectangular cross-section b×h and length L, resting on a foundation whose elasticity coefficient changes stepwise according to relation (5), i.e.,

Theoretical calculations were performed in the Mathematica™ (version 11.1 was used), environment using the NMinimize command to minimize the function describing the Rayleigh quotient (Gliński [44,45]). The independent variables in the minimization process were: the number of half-waves *m*, the asymmetry parameter *α*, and the sharpness parameter *μ*, which made it possible to determine the most probable buckling form and its corresponding critical force value.

The numerical simulations were carried out using the finite element method in the COSMOS/M 2.5 (Rusiński [46]) and Simulia Abaqus 2024 (Dassault Systèmes [47]) software, employing the Linear Buckling Analysis (LBA) modules. The FEM models included a bar with identical geometric, boundary conditions, material parameters and elastic support along its length, reproducing the stepwise change in the Winkler coefficient. The discretization density of the finite element mesh was determined based on preliminary analyses conducted with various sizes of elements. The results obtained using the adopted mesh were also verified against those computed for the same mesh but with different types of finite elements.

This made it possible to determine the first eigenvalue of the system, corresponding to the critical force initiating the loss of stability. The obtained results were used to assess the consistency between the theoretical model and the numerical solutions as well as to verify the influence of parameters $k_{1}$, $k_{2}$ and $\xi_{0}$ on the values of the critical force.

### 3.1. Example 1

In Example 1, an axially compressed bar hinged at both ends is analyzed. The bar has a rectangular cross-section b×h, length L, and rests on a two-part foundation whose elasticity coefficient changes stepwise (Figure 2). The input data are as follows:

bar length:

L=3100 mm,

bar cross-section:b=60 mm, h=6 mm,moment of inertia of the cross-section:

$I=bh^{3}/12={1080\;mm}^{4},$

Young’s modulus:$E=1.65\times 10^{5}\;MPa$,stiffness of the elastic medium:$k_{1}=2.50\;N/{mm}^{2}\;,{\;k}_{2}=1.10\;N/{mm}^{2}$,point of change in the stiffness of the medium:

$\xi_{0}=1/2.$



By performing a computational analysis, an estimate of the critical force was obtained

(11) $$F_{cr}=29.00\;kN,$$

and parameters describing the asymmetry and sharpness of the buckling form transition were determined

(12) m=9,  α=1.095,  μ=7.470. 

The obtained results form the basis for further analysis of the influence of model parameters on the nature of stability loss and the shape of the buckling form.

**Figure 3:**
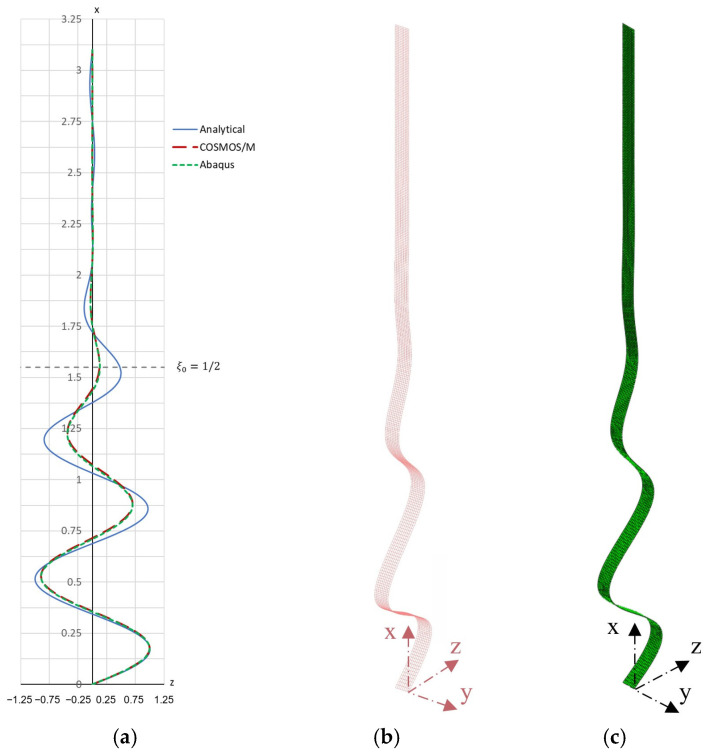
First buckling mode shapes of the bar for Example 1: (**a**) comparison of analytical and numerical solutions showing normalized displacements, (**b**) first buckling mode obtained using COSMOS/M 2.5, (**c**) first buckling mode obtained using Simulia Abaqus 2024.

Numerical simulations (linear buckling analysis—LBA) were also performed in order to determine the critical force and buckling form. The COSMOS/M system and the Simulia Abaqus system, both based on the finite element method (Rusiński [46]; Dassault Systèmes [47]), were used. In the COSMOS/M program, the bar in the form of a flat strip was discretized using quadrilateral finite elements SHELL4 from the shell element group. The elements were assigned appropriate values of the elastic foundation stiffness. The discrete model consisted of 3200 elements and approximately 20,000 degrees of freedom. A reference load of 1 kN was applied to the upper surface of the bar in the form of a distributed pressure. The boundary conditions were defined by constraining all translational displacements at the nodes of the lower end of the bar (hinged support) and by constraining the translational displacements *u_y_* and *u_z_* at the nodes of the upper end (loaded end with the possibility of longitudinal movement, $u_{x}\neq 0$). The adopted coordinate system is shown in Figure 3b. An analogous model was developed in the Simulia Abaqus program (Figure 3c). The bar was modeled using shell elements of type S4R (one integration point). According to the data, the elements were assigned elastic foundation support conditions. The boundary conditions were defined by constraining all translational displacements at the nodes of the lower end of the bar (hinged support) and by constraining the translational displacements *u_y_* and *u_z_* at the nodes of the upper end (loaded end with the possibility of longitudinal movement, $u_{x}\neq 0$). The discrete model consisted of 3104 elements and 21,006 degrees of freedom.

The method of bar modeling corresponds to the plate model. To make it consistent with the bar model adopted in the analytical solution, Poisson’s ratio was reduced to zero.

As a result of the performed linear buckling analysis (LBA), the critical force values obtained in the COSMOS/M and Simulia Abaqus programs were respectively $F_{cr}^{\left(C\right)}=28.61\;kN$ and $F_{cr}^{\left(A\right)}=28.595\;kN$ and the buckling form is shown in Figure 3b,c.

Both results were analyzed with respect to mesh density. The value of critical forces $F_{cr}^{\left(C\right)}=28.610\;kN$ was obtained for an average element size of 7.5 mm. It is worth mentioning that for an average element size of 15 mm $F_{cr}^{\left(C\right)}=28.640\;kN\;(only\;0.1\%\;higher$), it means that the mesh density is quite enough. The same tendency was observed in the case of the result obtained by the Simulia Abaqus program.

**Figure 4:**
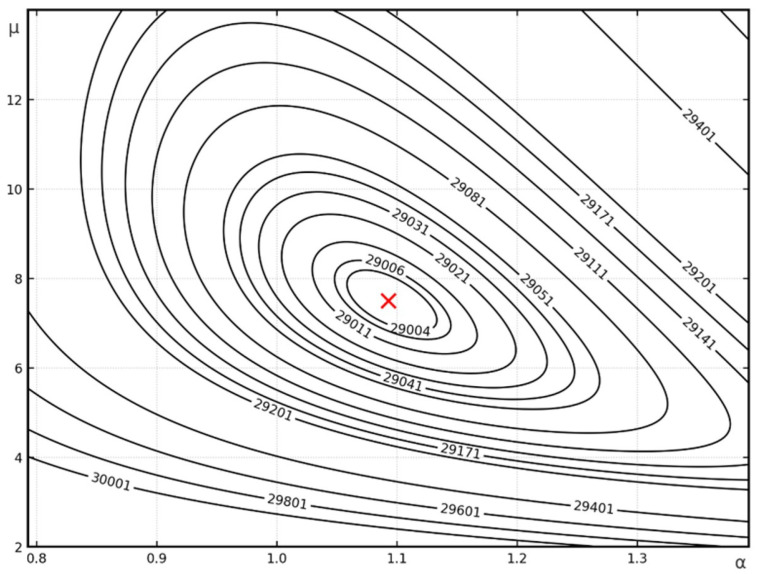
Contour map of the critical force for *m* = 9 for Example 1. The red cross indicates the absolute minimum of the function.

A graphical representation of the buckling form obtained analytically (for A=1 mm) is shown in Figure 3a, and the contour plan of the critical force in Figure 4.

### 3.2. Example 2

In Example 2, an axially compressed bar of the same shape is analyzed, but with different bar parameters. The input data are as follows:

bar length: 

L=1500 mm,

bar cross-section:b=50 mm, h=6 mm,moment of inertia of the cross-section:

$I=bh^{3}/12={900\;mm}^{4},$

Young’s modulus:$E=1.25\times 10^{5}\;MPa$,stiffness of the elastic medium:$k_{1}=0.65\;N/{mm}^{2}\;,k_{2}=2.20\;N/{mm}^{2}$,point of change in the stiffness of the medium:

$\xi_{0}=1/2.$



As before, the critical force values were determined numerically:

(13) $$F_{cr}=18.65\;kN,$$

and parameters describing the asymmetry and sharpness of the buckling form transition:

(14) m=4,  α =−1.106,  μ=5.775 

**Figure 5:**
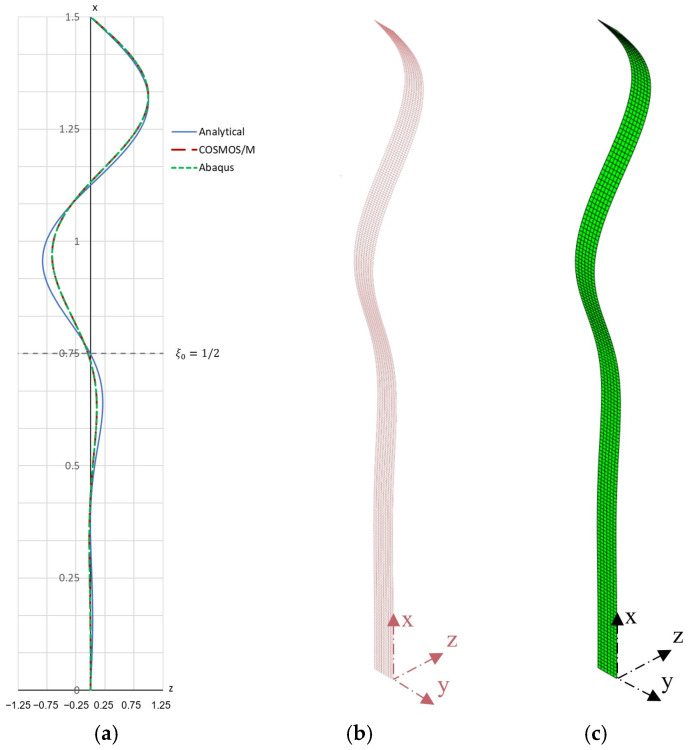
First buckling mode shapes of the bar for Example 2: (**a**) comparison of analytical and numerical solutions showing normalized displacements, (**b**) first buckling mode obtained using COSMOS/M 2.5, (**c**) first buckling mode obtained using Simulia Abaqus 2024.

In numerical buckling analyses similar to Example 1, performed in COSMOS/M and Simulia Abaqus, the critical force values $F_{cr}^{\left(C\right)}=18.34\;kN$ and $F_{cr}^{\left(A\right)}=18.335\;kN$ were obtained. The influence of the mesh density on the obtained results of the critical forces was very similar to in Example 1. The resulting buckling modes are shown in Figure 5b,c.

**Figure 6:**
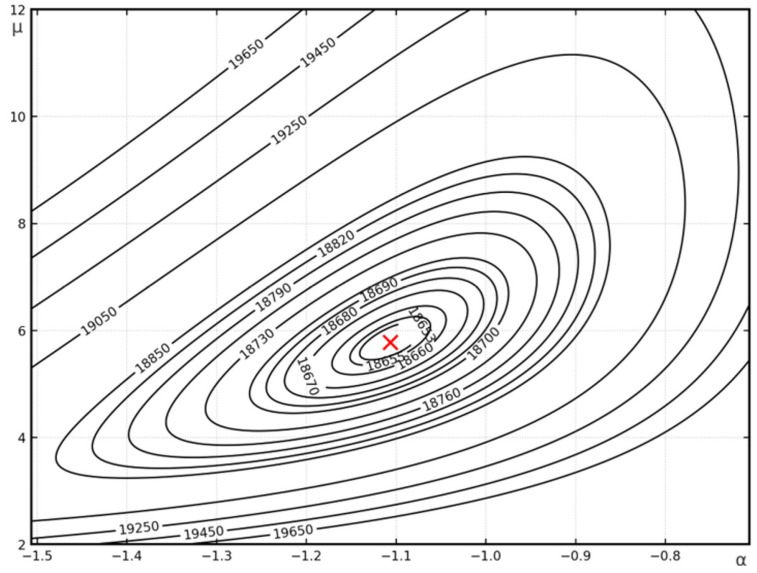
Contour map of the critical force for *m* = 4 for Example 2. The red cross indicates the absolute minimum of the function.

A graphical representation of the analytically obtained buckling mode (for A=1 mm) is shown in Figure 5a, and a contour plan of the critical force is shown in Figure 6.

### 3.3. Example 3

In Example 3, another variant of the axially loaded bar hinged at both ends is examined. The input data are as follows:

bar length:

L=2600 mm,

bar cross-section:b=75 mm, h=6 mm,moment of inertia of the cross-section:

$I=bh^{3}/12={781.25\;mm}^{4},$

Young’s modulus:$E=1.40\times 10^{5}\;MPa$,stiffness of the elastic medium:$k_{1}=1.50\;N/{mm}^{2}\;,k_{2}=2.15\;N/{mm}^{2}$,point of change in the stiffness of the medium:

$\xi_{0}=1/3.$



Unlike in the previous examples, the point of change in the foundation stiffness was shifted from the middle of the bar’s length to one-third of its length. Such a configuration makes it possible to examine the influence of asymmetrical distribution of zones with different stiffness on the form of stability loss and on the value of the critical force.

Analogously to the previous analyses, the calculations were carried out in the Mathematica™ environment using the Rayleigh quotient minimization procedure.

This made it possible to determine the value of the critical force

(15) $$F_{cr}=26.45\;kN,$$

and parameters describing the asymmetry and sharpness of the buckling form transition:

(16) m=9,  α =−1.105,  μ=6.488 

**Figure 7:**
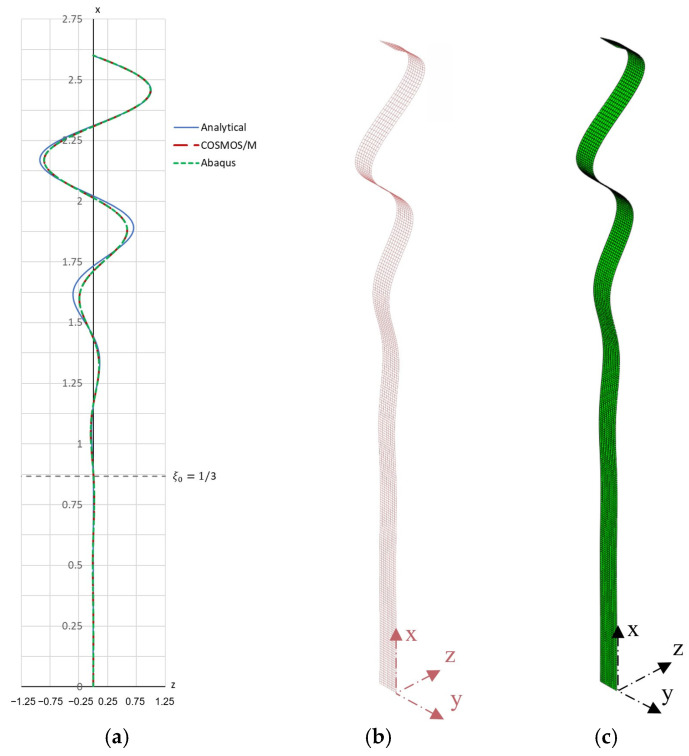
First buckling mode shapes of the bar for Example 3: (**a**) comparison of analytical and numerical solutions showing normalized displacements, (**b**) first buckling mode obtained using COSMOS/M 2.5, (**c**) first buckling mode obtained using Simulia Abaqus 2024.

In numerical buckling analyses, analogous to the previous examples and performed in the COSMOS/M and Simulia Abaqus programs, the obtained values of the critical force were $F_{cr}^{\left(C\right)}=26.40\;kN$ and $F_{cr}^{\left(A\right)}=26.379\;kN$. The influence of the mesh density on the obtained results of the critical forces was very similar to in Example 1. The obtained buckling forms are shown in Figure 7b,c.

**Figure 8:**
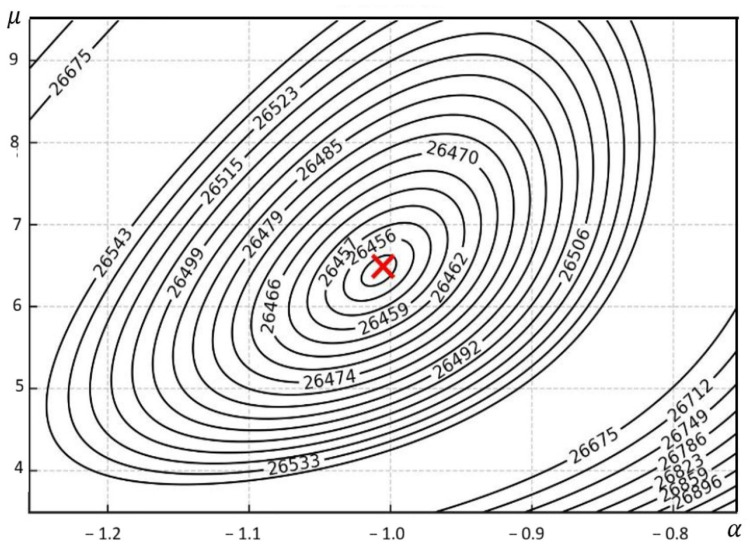
Contour map of the critical force for *m* = 9 for Example 3. The red cross indicates the absolute minimum of the function.

The graphical representation of the buckling form obtained analytically (for A = 1 mm) is shown in Figure 7a, and the contour plot of the critical force in Figure 8.

The analytical and numerical solutions obtained were compared with the COSMOS/M results (Table 1). In the examples considered, the discrepancy (Δ) between the numerical solutions did not exceed 0.1%. The findings also indicate a slight overestimation of the critical force obtained by the proposed analytical approach—up to 1.7%—which can be regarded as acceptable.

### 3.4. Example 4

In this example, the bar from Example 1 was analyzed for a wide range of stiffness ratios $k_{1}/k_{2}$ from 0.1 to 10, with $k_{1}=2.5$ as a constant value. It is important to note that the numerical simulations were performed under three-dimensional conditions. In the considered case, for stiffness ratios $k_{1}/k_{2}<1$, these conditions permitted buckling in the perpendicular (x−y) plane. To ensure buckling only within the (x−z) plane, translations in the y-direction were constrained.

The analytical and numerical solutions obtained were compared with the COSMOS/M results (Table 2). In the examples considered, the discrepancy (Δ) between the numerical solutions did not exceed 0.1%. The results also indicate some tendency to overestimation of the critical load predicted by the proposed analytical approach for large stiffness differences (0.3>k1/k2>5) up to 5%, which can still be regarded as acceptable from an engineering point of view.

### 3.5. Example 5

Unlike in the previous examples, in this case, a constant foundation stiffness was assumed along the entire rod. This setup makes it possible to examine the influence of uniform stiffness on the buckling mode and on the value of the critical load. As in the earlier analyses, the computations were carried out in Mathematica™, using a procedure based on Rayleigh quotient minimization. The input data are as follows:
bar length:L=2400 mm,bar cross-section:b=55 mm, h=7 mm,moment of inertia of the cross-section:$I=bh^{3}/12={1572.08\;mm}^{4},$Young’s modulus:$E=0.75\times 10^{5}\;MPa$,constant stiffness of the elastic medium:$k_{1}=k_{2}=k=1.70\;N/{mm}^{2}$,point of change in the stiffness of the medium:$\xi_{0}=0.$

In this case, the critical load is as follows:

$$F_{cr}=28.43\;kN,$$

while the parameters governing the asymmetry and sharpness of the buckling-mode transition are as follows:

$$m=8,\alpha =-4.08\cdot 10^{-7}\approx 0,\mu =-0.0325\approx 0$$

This shows that the critical load occurs for a practically zero value of parameter α, which reduces the buckling mode (8) to w(ξ)=Asin (mπξ), and the minimization of functional (9) reduces to finding the minimum of

$$F(m)=EI{\left(\frac{m\pi }{L}\right)}^{2}+\frac{kL^{2}}{(m\pi {)}^{2}}$$

which ultimately leads to the classical formula $F_{cr}=2\sqrt{EIk},$ and then, $F_{cr}=2\sqrt{EIk}\approx 28.4\;\mathrm{kN},$ which is fully consistent with the value obtained through the proposed approach.

### 3.6. Example 6

This example assumes zero foundation stiffness along the entire rod length and examines its influence on the buckling mode and the critical load. As before, the analysis was performed in Mathematica™, using the Rayleigh-quotient minimization procedure. The input data are as follows:
bar length: L=1600 mm,bar cross-section:b=57 mm, h=6 mm,moment of inertia of the cross-section:$I=bh^{3}/12={1026.00\;mm}^{4},$Young’s modulus:$E=1.35\times 10^{5}\;MPa$,stiffness of the elastic medium:$k_{1}=k_{2}=0.0\;N/{mm}^{2}$,point of change in the stiffness of the medium: $\xi_{0}=0.$

In this case, the critical load is as follows:

$$F_{cr}=533.9\;N,$$

while the parameters governing the asymmetry and sharpness of the buckling-mode transition are as follows:

$$m=1,\;\;\;\;\;\alpha =4.25,\;\;\;\;\;\mu =6.8\cdot 10^{-10}\approx 0$$

This again shows that the critical load occurs for a practically zero value of the parameter μ, which reduces the buckling mode (8) to w(ξ)=Asin(πξ), and the minimization of functional (9) reduces to the classical Euler formula:

$$F_{cr}=\frac{\pi^{2}EI}{L^{2}},$$

and then, $F_{cr}=\pi^{2}EI/L^{2}\approx 534.00\;N,$ which is fully consistent with the value obtained by the proposed approach.

## 4. Discussion and Conclusions

The present study introduces three essential new contributions to the literature, pertaining both to methodology and to the interpretation of the buckling behavior of a beam resting on a foundation with a stepwise variation in stiffness. First, a novel three-parameter buckling mode is proposed, incorporating the number of half-waves m, an asymmetry parameter α and a transition-sharpness parameter μ. This function has been specifically designed for systems featuring a discontinuity in the Winkler modulus, for which classical sinusoidal shapes fail to reproduce the actual deformation pattern. It enables not only the adjustment of the number of half-waves and the degree of asymmetry, but also the modeling of either a smooth or a sharp curvature transition at the stiffness jump –phenomena not previously addressed within an analytical or energy-based framework.

Second, a multi-parameter version of the Rayleigh–Ritz method is employed, in which the energy quotient is minimized simultaneously with respect to the three parameters of the trial function. This approach allows the optimal buckling shape to be determined automatically for any given location of the stiffness discontinuity and any stiffness contrast, without prescribing the deformation shape in advance. The trial function satisfies the kinematic boundary conditions of the system but does not enforce the other conditions (zero bending moment at the ends). In accordance with Rayleigh’s method, this leads to a controlled upper bound of the critical load, resulting from a local overestimation of the bending strain energy near the beam ends. The study provides a complete variational justification of this effect, together with an interpretation of the error sources.

Third, a semi-analytical procedure is developed for determining the critical load of a beam on a Winkler foundation with a single stiffness discontinuity—a configuration commonly encountered in engineering applications (e.g., layered soil–structure interaction, sheet-pile walls in stratified ground, as well as aircraft and marine structural elements supported by non-uniform substrates). Until now, the literature has lacked a clear description of the influence of a stepwise change in foundation stiffness on the critical load and the corresponding buckling mode. The proposed method enables the analysis of any discontinuity location and any stiffness ratio $k_{1}/k_{2}$. The obtained results show excellent agreement with independent numerical simulations in COSMOS/M and ABAQUS, with relative differences not exceeding 1.7%, provided that the following condition is fulfilled: $0.3<k_{1}/k_{2}<5$. Otherwise, the error rises to nearly 5%, which is acceptable from an engineering point of view.

From the point of view of structural mechanics, the study demonstrates that a stepwise change in foundation stiffness leads to buckling modes that strongly localize within the region of lower stiffness, regardless of the position of the discontinuity. This indicates the limited effectiveness of further stiffening the segment with a higher value of k, as the buckling mode inevitably shifts toward the weaker part of the foundation. The parameters α, m, and μ allow an accurate description of the localization of the mode shape, the asymmetry of deflection, and the nature of the transition between the beam segments supported by foundations of different stiffness. The employed energy method provides reliable estimates of the critical load and enables both qualitative and quantitative assessment of the influence of the stiffness discontinuity.

Thus, variations in foundation stiffness may be regarded as a purposeful design variable, allowing engineers to control the buckling resistance of structural elements interacting with an elastic medium. The developed method forms a basis for further studies involving geometric and material nonlinearities, post-critical behavior, and foundations with multiple parameters (e.g., Pasternak-type models). The results confirm that controlled variability of foundation stiffness can serve as an effective tool for optimizing the stability of structures interacting with elastic foundations.

The developed model was verified numerically in COSMOS/M and ABAQUS, and the results showed very good agreement with the theoretical predictions—the relative differences did not exceed 1.7%. The analysis confirmed that the stepwise change in foundation stiffness significantly affects both the critical load and the buckling mode: transverse deformations concentrate in the regions with lower foundation stiffness. The proposed method can be applied for the preliminary assessment of the load-carrying capacity of engineering structures interacting with non-homogeneous soil, such as steel sheet piles. This method forms a basis for further studies of members embedded in media with more complex elastic characteristics.

The results confirm the effectiveness of the energy method, in which the assumed deformation form is described by three independent parameters, determined by minimizing the expression for the critical load.

## Figures and Tables

**Table 1:** Results of critical force calculations.

Bar No.	Analytical		COSMOS/M	Simulia Abaqus	
	$F_{cr},kN$	Δ,%	$F_{cr},kN$	$F_{cr},kN$	Δ,%
1	29.00	+1.36	28.61	28.60	−0.05
2	18.65	+1.69	18.34	18.34	−0.02
3	26.45	+0.18	26.40	26.38	−0.07

**Table 2:** Results of critical force calculations for different stiffness ratios $k_{1}/k_{2}$ for Example 1.

$k_{1}/k_{2}$	$k_{2}$	Analytical		COSMOS/M	Simulia Abaqus	
		$F_{cr},kN$	Δ,%	$F_{cr},kN$	$F_{cr},kN$	Δ,%
0.1	25.00	44.78	4.32	42.93	42.89	−0.09
0.3	8.33	43.85	2.24	42.89	42.86	−0.07
0.5	5.00	43.28	1.01	42.85	42.81	−0.09
1	2.50	43.20	2.13	42.3	42.26	−0.09
2	1.25	30.74	1.02	30.43	30.42	−0.03
5	0.50	20.41	4.68	19.5	19.49	−0.05
10	0.25	14.57	4.74	13.91	13.9	−0.07

## Data Availability

The original contributions presented in this study are included in the article. Further inquiries can be directed to the corresponding author.

## Nomenclature

The following symbols and notations are used in this manuscript:

A	Amplitude of buckling mode
α	Asymmetry parameter (sign and strength of buckling location)
b×h	Bar cross-section
E	Young’s modulus
$F_{cr}$	Critical compressive load
k(x)	Local foundation stiffness
ξ	Normalized variable
$\xi_{0}$	Point of change in the stiffness of the medium
L	Bar length
m	Number of half-waves along the length of the bar (integer)
M(x)	Bending moment
γ, μ	Transition sharpness parameters
$U_{k}$	Elastic energy of the elastic substrate
$U_{g}$	Bending strain energy of the bar
U	Total potential energy of the system
V	Work performed by the axial force
